# Editorial: Crosstalk between computational medicine and neuroscience in healthcare

**DOI:** 10.3389/fnins.2023.1333227

**Published:** 2023-11-30

**Authors:** Zhijin Wang, Philippe Fournier-Viger, Yu-Ping Wang, Yonggang Fu

**Affiliations:** ^1^College of Computer Engineering, Jimei University, Xiamen, China; ^2^College of Computer Science and Software Engineering, Big Data Institute, Shenzhen University, Shenzhen, China; ^3^School of Science and Engineering and School of Public Health and Tropical Medicine, Tulane University, New Orleans, LA, United States

**Keywords:** computational medicine, neuroscience, healthcare, artificial intelligence, health

Advances in neuroscience and computational technologies are transforming medical practices, enabling more tailored treatments for complex neurological disorders. The integration of Artificial Intelligence (AI) is important in providing enhanced efficiency and cost reduction in healthcare services. The expansion of extensive datasets, coupled with increased computational capabilities, is dismantling previous obstacles in analyzing medical and neurological data. However, conventional medical approaches, which are based on static expert knowledge, frequently fall short of adequately interpreting the dynamic relationships among biomarkers, disease progression, and treatment outcomes, consequently hindering the refinement of clinical decisions.

This Research Topic is designed to bring together experts in computational medicine and neuroscience to tackle healthcare challenges with a multidisciplinary approach. The aim is to utilize the increasing abundance of neurological data and computational resources to transition from reactive to proactive, individualized healthcare strategies. Numerous high-quality submissions were submitted to this Research Topic.

All submissions were subject to peer review by at least two researchers in the relevant domains. The accepted articles in this Research Topic are briefly introduced below.

The paper entitled “*Golgi_DF: Golgi proteins classification with deep forest*” by Bao et al. presented the Gogli_DF model for classifying Golgi proteins. This approach utilizes a deep forest algorithm enhanced by advanced feature extraction and sample balancing techniques. It employs dimensionality reduction, convolutional and LSTM networks for robust feature fusion. The approach signifies a substantial improvement in predictive accuracy for Golgi protein classification.

The paper, titled “*Heterogeneous temporal representation for diabetic blood glucose prediction*” by Hunag et al. proposed a predictive model HETER for blood glucose levels in multiple patients with diabetes that addresses data heterogeneity and uncertainty. The model uses subsequence repetition and graph neural networks to effectively process and analyze variable-length CGM data sequences and outperforms traditional methods, providing robust and generalizable predictions across diverse patient dataset. Experimental results show the effectiveness of the proposed method.

The paper titled “*Early prediction of atherosclerosis diagnosis with medical ambient intelligence*” by Yang et al. designed a Medical Ambient Intelligence System (MAIS) for the early detection of atherosclerosis. The system integrates the entire clinical process from data collection to decision visualization, enhancing the diagnosis task's performance. Moreover, the MAIS and SEC model together demonstrate superior performance compared to existing methods and identify Vitamin B12 as a key biomarker for early-stage atherosclerosis detection. This also shows the promising of medical healthcare system in integrating smart diagnosis techniques.

The paper “*A face image classification method of autistic children based on the two-phase transfer learning*” by Li et al. proposed an improved facial recognition algorithm to screen for autism in children using mobile-friendly deep learning models. By integrating two transfer learning phases and multiple classifiers, the model aims to increase detection accuracy. This approach offers a quick, resource-efficient method for early autism identification.

Editors hope that this Research Topic will serve as a wellspring of both inspiration and substantive insights, contributing to advancing applied intelligence within the health and healthcare sectors across theoretical and practical dimensions. This Research Topic provides numerous opportunities for researchers and practitioners to further utilize and expand upon the presented research. The editors sincerely appreciate the effort and dedication of all contributors to this Research Topic.

## Author contributions

ZW: Writing—original draft. PF-V: Writing—review & editing. Y-PW: Writing—review & editing. YF: Writing—review & editing.

